# Peptide-Modified Nano-Bioactive Glass for Targeted Immobilization of Native VEGF

**DOI:** 10.1021/acsami.1c21378

**Published:** 2022-01-18

**Authors:** Matthias Schumacher, Pamela Habibović, Sabine van Rijt

**Affiliations:** Department of Instructive Biomaterials Engineering, MERLN Institute for Technology-Inspired Regenerative Medicine, Maastricht University, 6229 ER, Maastricht, Netherlands

**Keywords:** nanoparticles, peptide functionalization, bioactive glass, VEGF, angiogenesis, vascularization, bone regeneration, biomaterial

## Abstract

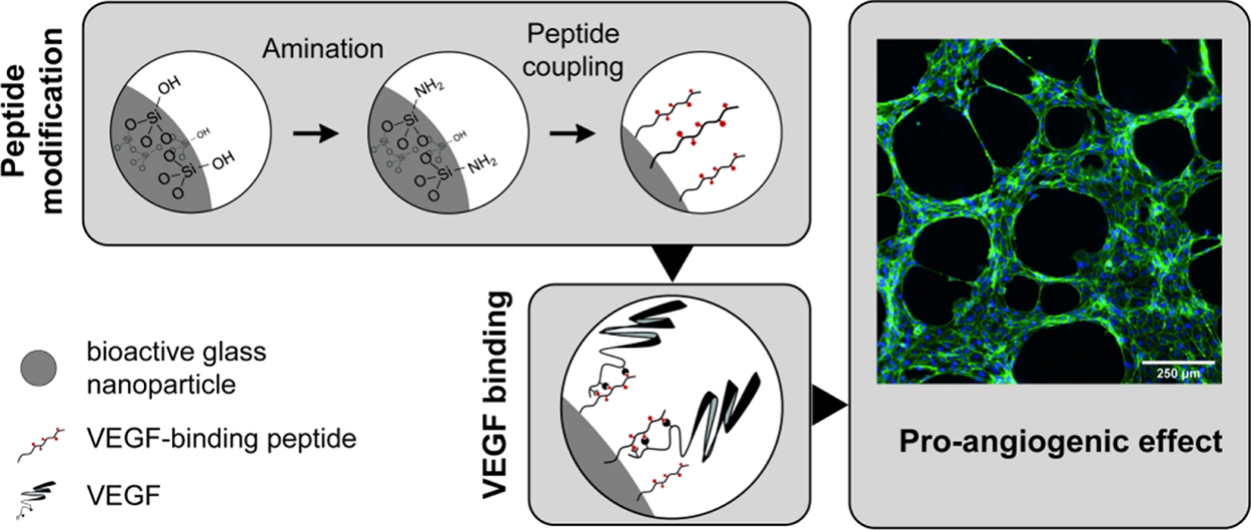

A limiting factor in large bone defect regeneration is the slow and disorganized formation of a functional vascular network in the defect area, often resulting in delayed healing or implant failure. To overcome this, strategies that induce angiogenic processes should be combined with potent bone graft substitutes in new bone regeneration approaches. To this end, we describe a unique approach to immobilize the pro-angiogenic growth factor VEGF_165_ in its native state on the surface of nanosized bioactive glass particles (nBGs) via a binding peptide (PR1P). We demonstrate that covalent coupling of the peptide to amine functional groups grafted on the nBG surface allows immobilization of VEGF with high efficiency and specificity. The amount of coupled peptide could be controlled by varying amine density, which eventually allows tailoring the amount of bound VEGF within a physiologically effective range. In vitro analysis of endothelial cell tube formation in response to VEGF-carrying nBG confirmed that the biological activity of VEGF is not compromised by the immobilization. Instead, comparable angiogenic stimulation was found for lower doses of immobilized VEGF compared to exogenously added VEGF. The described system, for the first time, employs a binding peptide for growth factor immobilization on bioactive glass nanoparticles and represents a promising strategy to overcome the problem of insufficient neovascularization in large bone defect regeneration.

## Introduction

Angiogenesis plays a pivotal role in tissue formation and regeneration, and therefore, it emerged as a target for regenerative medicine over the past years. Today, the insufficient or imbalanced development of a functional vascular network that ensures nutrient and oxygen supply within larger defect areas remains a limiting factor in clinical regenerative medicine, including bone regeneration.^1^ As bone is a highly vascularized tissue, regenerative approaches must not neglect the need to promote the formation of a functional vascular network while focusing on stimulating both new bone formation and angiogenesis in a coordinated manner.^2^ To achieve this, strategies that induce pro-angiogenic signaling pathways during the initial stages of bone healing^3^ and thereby control angiogenic processes (therapeutic angiogenesis, TA) should be combined with existing bone regeneration approaches.^4−6^

A central tool in TA are pro-angiogenic agents such as growth factors (GF). These highly potent signaling molecules can regulate cell migration,^7,8^ proliferation and survival,^8−10^ structural organization,^11,12^ and differentiation.^8,13^ The vascular endothelial growth factor (VEGF) is the most prominent regulator of angiogenesis and neovascularization.^14^ However, the nontargeted, systemic administration of any GF to promote local tissue regeneration bears the risk for off-site effects, applying supraphysiological concentrations and loss of biological activity due to protein conformation changes or proteolytic degradation.^5^ Moreover, VEGF also plays a role in cancer-related angiogenesis,^15^ diabetes,^16^ atherosclerosis,^17^ and other pathological states. Therefore, controlling dosage and spatial distribution is of utmost importance. This explains why targeted administration with the help of suitable carriers is considered a superior approach to stimulate the different stages of tissue regeneration, including angiogenesis, with the help of GFs.^18^

A plethora of strategies has been proposed for targeted GF administration.^18−20^ In bone regeneration, systems to immobilize osteogenic and chondrogenic GFs (bone morphogenetic proteins BMP-2 and BMP-7) as well as GFs involved in cell recruitment and proliferation (platelet-derived growth factor PDGF and BMP-6) have been developed to enhance the osteogenic potential of synthetic bone graft substitutes.^21^ Delivery of VEGF to stimulate vasculature formation has been described using hydrogels,^22,23^ polymeric microspheres,^24−27^ bioceramics, (mesoporous) bioactive glasses,^28−31^ and mesoporous silica nanoparticles.^32^ However, in most reported cases, the immobilization of GFs (including VEGF) on carriers is based on either physical entrapment or electrostatic interactions. Physical entrapment requires tight control of the degradation of the encapsulating material to avoid burst release kinetics,^33^ and the choice of encapsulating material and solvents during encapsulation may affect protein activity.^34^ Electrostatic interactions, on the other hand, are poorly controllable in terms of adsorption and release kinetics and may deteriorate GF protein conformation and activity as recently reviewed by Zheng et al.^35^ An alternative approach is to immobilize GFs permanently on material surfaces via covalent coupling.^36,37^ Although several studies have demonstrated a pro-angiogenic effect of VEGF covalently coupled to (bio)polymers,^38−40^ this strategy can also lead to changes in growth factor conformation or manipulation in their functional regions, resulting in a loss of biological activity.^41^ Therefore, immobilization strategies that allow GF immobilization in the native state would allow designing novel carrier systems with higher biological activity.

To achieve such immobilization, we propose ternary bioactive glass nanoparticles (nBGs) decorated with peptides that can bind specific GFs in their native state with high specificity. Aiming to add pro-angiogenic functionality to nBGs, we use a peptide (PR1P) derived from the VEGF-binding domain of prominin-1.^42,43^ PR1P binds VEGF_165_ (but not other isoforms), and previous studies have shown that binding to PR1P enhances the biological activity^42^ and stability^44^ of VEGF. Thus, the proposed approach is expected to allow sustained immobilization of VEGF with unimpaired biological activity. To date, no attempts have been published to immobilize VEGF on nanosized bioactive glass particles (nBGs), although nBGs have been identified as potent vehicles for various drugs and other bioactive factors^35,45,46^ and a synergistic effect of VEGF and Si ions released from silica-based materials has been shown.^32,47,48^ As all bioactive glasses, nBGs show high, composition-dependent surface reactivity (bioactivity) that stimulates in vivo bone bonding, have excellent biocompatibility, and show controlled degradation.^49^ These characteristics have been utilized in bone regeneration by formulating composites from nBGs and biopolymers^50^ and hydrogels.^51^

Here, we aim to combine a novel TA strategy with a highly potent biomaterial for bone regeneration. We covalently coupled a peptide sequence (PR1P),^42^ which binds the vascular endothelial growth factor (VEGF_165_)^52^ with high selectivity and affinity to nBG particles. The obtained construct was tested with respect to the functionalization degree, VEGF binding efficiency, and specificity. In addition, we used an in vitro endothelial cell tubule formation assay to test the biological activity and stability of immobilized VEGF*.*

## Results and Discussion

### nBG Synthesis and Peptide Surface Modification

**Figure 1:**
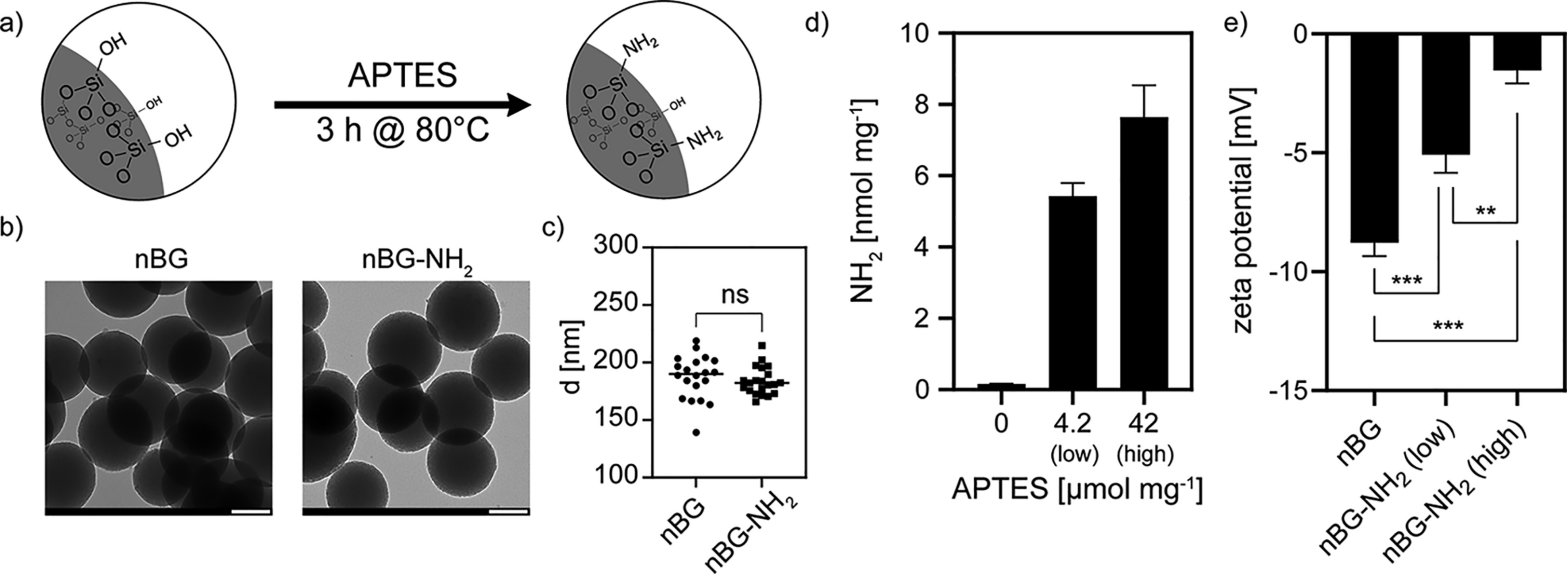
(a) nBG surface modification with amines. (b) TEM micrographs of as-synthesized (nBG) and amine-modified nBG (nBG-NH_2_); scale bar, 100 nm. (c–e) Particle size (c), amine density (FITC-NHS binding assay) (d), and zeta potential (e) at different processing steps (*P < 0.033, **P < 0.002, ***P < 0.001).

**Figure 2:**
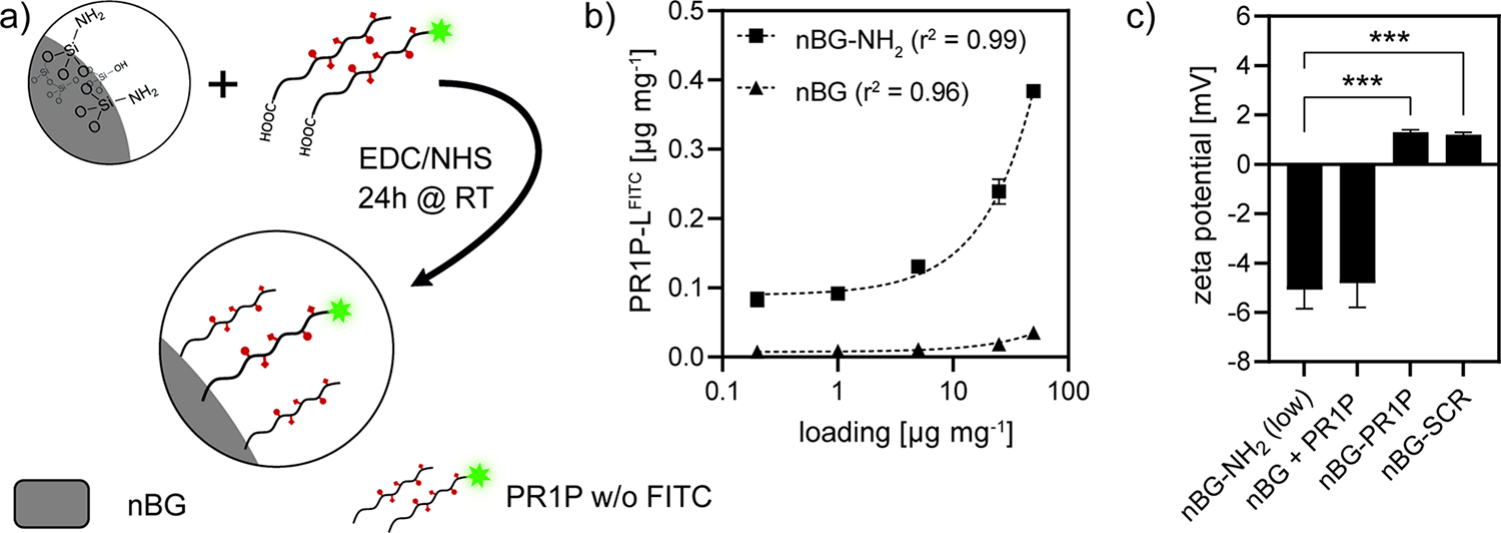
(a) Peptide functionalization of nBG-NH_2_ particles. (b) Quantitative evaluation of PR1P functionalization of nBG-NH_2_ using different peptide/nBG ratios by fluorescence. (c) Change of zeta potential during peptide functionalization (SCR, scrambled PR1P; *P < 0.033, **P < 0.002, ***P < 0.001).

nBG particles were synthesized using a base-catalyzed sol–gel process using tetraethylorthosilicate, calcium nitrate tetrahydrate, and triethylphosphate as Si, Ca, and P sources, respectively. Methanol was used as solvent to ensure stoichiometric hydrolysis of TEOS and TEP as proposed by de Oliviera et al.^53^ Subsequently, the material was calcined at 600 °C. Nominal glass composition was set to SiO_2_/CaO/P_2_O_5_ = 80/15/5 mol % according to earlier studies suggesting that sol–gel derived glasses of such composition exhibit high bioactivity and moderate degradation rates, which are considered advantageous for bone regeneration applications.^54,55^ Surface modification of nBG with amine groups was performed via postgrafting with 3-aminopropyl-triethoxysilane (APTES) in ethanol based on a protocol described by Zaharudin et al.^56^ (Figure 1a). The APTES-to-nBG ratio was varied to achieve different degrees of surface modification and thereby allow control over the number of binding moieties for subsequent peptide conjugation. Finally, a 12-mer peptide sequence derived from the transmembrane glycoprotein prominin-1 (PR1P: DRVQRQTTTVVA^43^) modified with a 8-mer polyglycine at the C terminus (DRVQRQTTTVVAGGGGGGGG) as a spacer between the functional (VEGF-binding) region of the peptide and the particle surface was covalently coupled to the nBG surface through amide coupling after EDC/NHS activation of the peptide (Figure 2a).

### Characterization of the Immobilization System

Transmission electron microscopy (TEM) was employed to assess nBG size and shape after synthesis and to monitor changes in morphology over the subsequent surface modifications with amines (Figure 1b). Material synthesis yielded uniform, round-shaped BG particles with an average size of *d*_nBG_ = 187.3 ± 19.0 nm as determined by TEM (Figure 1c). nBG was fully amorphous as confirmed by XRD (Figure S1). No indication of calcium phosphate crystallization during nBG synthesis was found. During postgrafting with APTES, amine functional groups (-NH_2_) were covalently bonded to silanol (Si–OH) groups on the nBG surface. Neither particle morphology nor size (*d*_nBG-NH2_ = 183.8 ± 12.0 nm) significantly changed during amine surface modification (Figure 1b,c). By varying the APTES/nBG ratios (4.2 and 42 μmol mg^–1^), increasing amine concentrations of 5.4 and 7.6 nmol mg^–1^ on the nBG could be obtained, respectively (Figure 1d), as assessed quantitatively using fluorescent labeling with NHS-FITC (Figure 1c).^57^ Since the density of the amines depended on the APTES/nBG ratio during modification, the amount of reactive moieties for the subsequent functionalization of the particles with peptides could be controlled. Surface amination was further apparent from zeta potential measurements (Figure 1e). Amine modification shifted the zeta potential more positively with the increasing APTES/nBG ratio. In contrast to the as-synthesized nBG, which exhibited a surface zeta potential of −8.7 ± 0.6 mV in PBS, a significantly more positive zeta potential of −5.1 ± 0.7 mV and – 1.5 ± 0.5 mV was found for nBG-NH_2_ treated with increasing amounts of APTES, reflecting the presence of positively charged amine groups on the particle surface.^56^ For subsequent peptide coupling experiments, the APTES/nBG ratio was fixed at 4.2 μmol mg^–1^.

An FITC-tagged version of the PR1P peptide (PR1P^FITC^) was used to monitor functionalization quantitatively. We found that the amount of peptide conjugated to nBG-NH_2_ depended on the ratio of peptide per nBG (w/w) during amide coupling. Interestingly, this dependency was almost linear (*r*^2^ = 0.9904; Figure 2b). This enables controlling peptide functionalization density via a second mechanism independent of the amine functional density (fixed at 4.2 nmol mg^–1^). A maximum functionalization of 0.38 μg mg^–1^ PR1P on nBG was achieved (loading ratio of 50 μg mg^–1^ peptide/nBG). Although BG surfaces in general can absorb significant quantities of proteins,^35^ the amount of PR1P that adsorbed nonspecifically to non-amine-modified nBG was more than 10 times lower than the efficiency of amide coupling (Figure 2b). Successful peptide conjugation also shifted the zeta potential toward positive values (nBG-PR1P: 1.3 ± 0.1 mV; nBG-L-SCR: 1.2 ± 0.1 mV*, P* < 0.033; Figure 2c).

### VEGF Immobilization Efficiency, Stability, and Specificity

**Figure 3:**
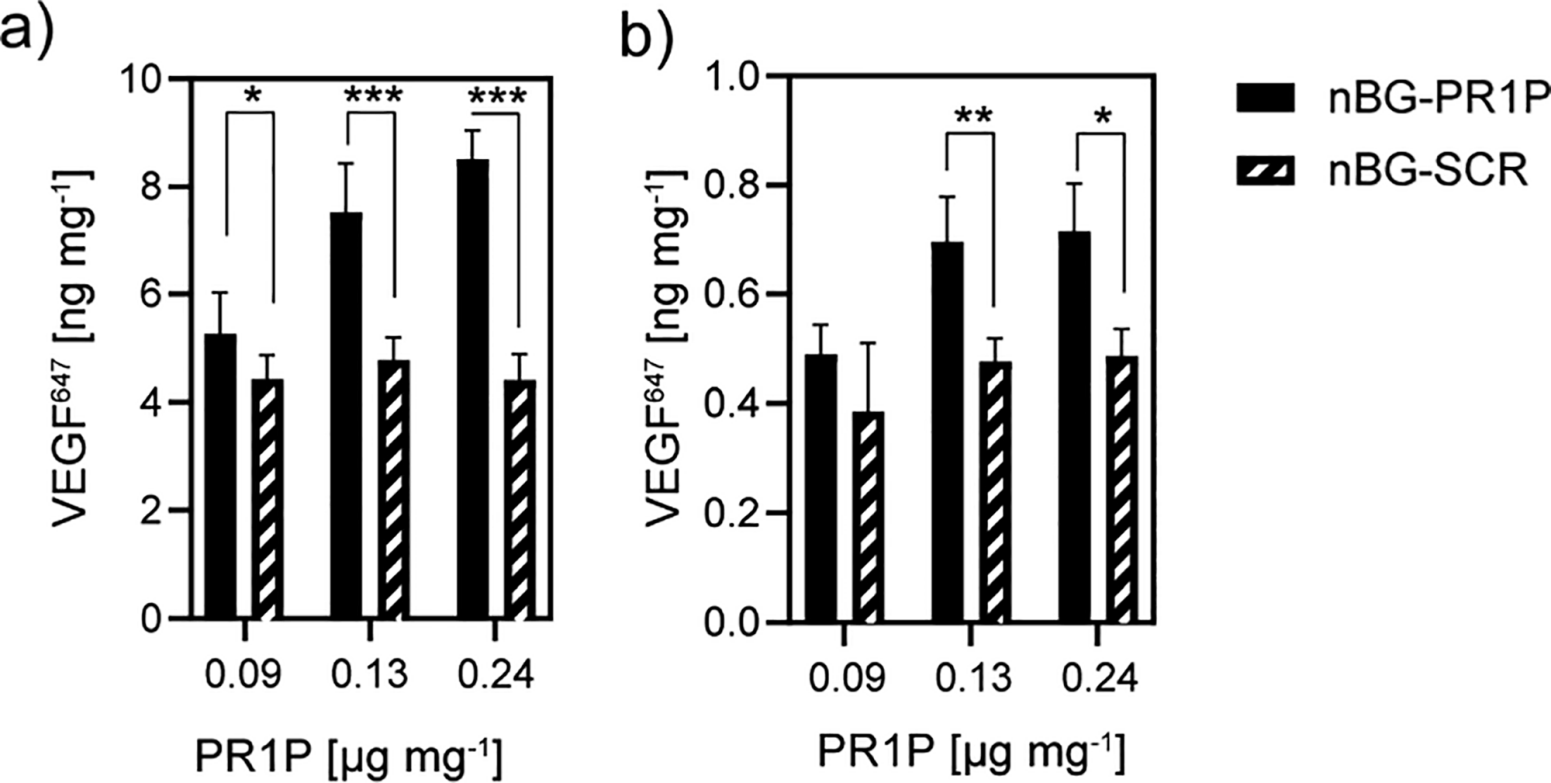
Quantitative analysis of VEGF binding to nBG particles functionalized with increasing amounts of PR1P or a scrambled version of the same sequence (SCR) via immersion in solutions with 2 μg mL^–1^ (a) or 0.2 μg mL^–1^ VEGF (b) for 3 h (*P < 0.033, **P < 0.002, ***P < 0.001).

Human recombinant VEGF protein binding to PR1P-functionalized nBG was facilitated by immersion in 2 or 0.2 μg mg^–1^ VEGF-containing buffer. To minimize degradation of the VEGF, which has limited half-life in solution,^58^ we chose a shorter loading time (3 h) compared to other studies using nanocarriers that usually performed loading for at least 12–24 h.^29,59,60^ Both the degree of functionalization and VEGF concentration in the immersion buffer were varied. Quantitative assessment of binding using fluorescent-tagged VEGF^647^ revealed that binding depended on the degree of PR1P functionalization of nBG and VEGF concentration in the immersion buffer (Figure 3). A maximum binding efficiency of up to 84.7% was found for particles functionalized with 0.24 μg mg^–1^ PR1P immersed in buffer containing 2 μg mL^–1^ VEGF; however, no significant increase in binding efficiency was found compared to nBG functionalized with 0.13 μg mg^–1^ PR1P. Therefore, for further experiments, functionalization was fixed to 5 μg mg^–1^ PR1P.

In their original study on PR1P, Adini et al.^42^ described that minor changes in the PR1P amino acid sequence drastically reduced the VEGF-binding effect of the PR1P peptide. We therefore used nBG particles functionalized with a scrambled version of the PR1P peptide (nBG-SCR; see Table 1) as a control to discriminate specific PR1P/VEGF binding from nonspecific protein–protein interactions. The binding efficiency to nBG-PR1P, which was measured directly after resuspension of sedimented nBG-PR1P/VEGF^647^ and nBG-SCR/VEGF^647^ in fresh buffer without additional washing, was about 30% higher compared to nBG-SCR.

**Table 1:** Amino Acid Sequence of VEGF-Binding Peptide (PR1P) Complemented with an N-Terminal Spacer and a Scrambled Version Comprising the Same Amino Acids (SCR)

label	sequence
PR1P	DRVQRQTTTVVAGGGGGGGG
SCR	QRDVAVTRTVQTGGGGGGGG

**Figure 4:**
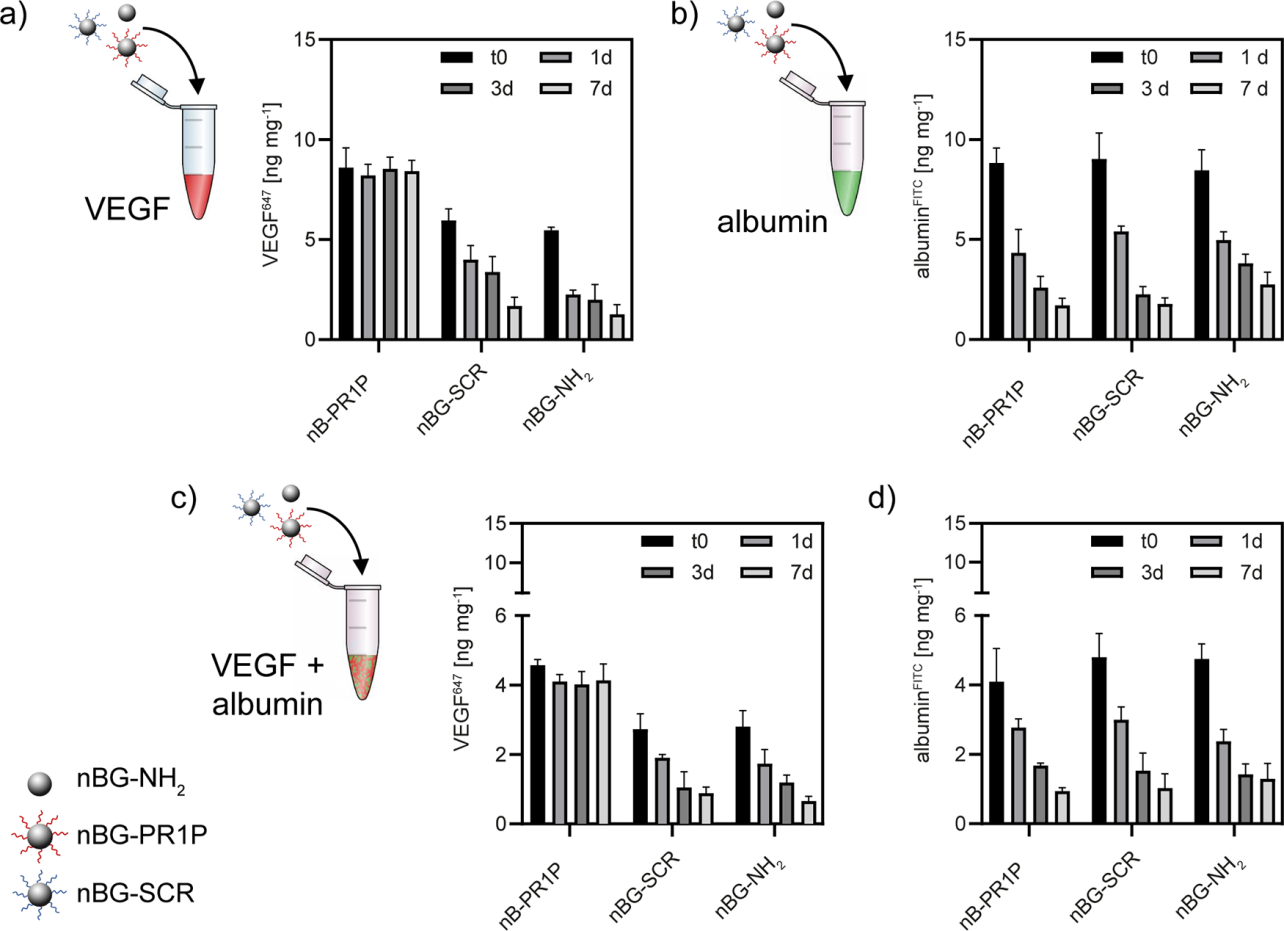
Binding of VEGF (labeled with Alexa Fluor 647) to nBG particles functionalized with 0.13 μg mg^–1^ PR1P, a scrambled peptide (SRC) and without functionalization (nBG-NH_2_): specific binding through immersion in VEGF-containing buffer for 3 h and stability of the formed complex over 7 days (a) vs nonspecific binding of (FITC-labeled) albumin and stability of the complex (b). Competitive binding of VEGF (c) and albumin (d) from a 1:1 protein mix and release over 7 days. Measurements were performed in suspension.

The stability of the nBG-PR1P/VEGF^647^ complex was subsequently studied using nBGs functionalized with 5 μg mg^–1^ PR1P and exposed to a buffer containing 2 μg mL^–1^ VEGF. No significant release of VEGF was observed despite constant agitation and multiple washing steps at each analysis time point (Figure 4a). With respect to the cascaded events during bone defect healing where the onset of angiogenesis happens between day 5 and 10, the stability of the complex appears sufficient. At the same time, nonspecifically bound VEGF was released from nBG-SCR and the nBG-NH_2_ control over the 7 day period. These findings demonstrate that the affinity of PR1P to bind VEGF^43^ was preserved upon coupling the peptide to the nBG surface and was stable. Next, nonspecific protein binding to (functionalized) nBG was studied by exposing particles to 2 μg mL^–1^ bovine serum albumin (BSA) solutions. Earlier studies have shown increased absorption of BSA to silica-glass surfaces upon amine surface modification,^61^ and indeed, high BSA binding was observed after initial exposure. No influence of the material functionalization was apparent (Figure 4b). Following the same 7 day treatment as described for the VEGF-treated samples above, BSA binding significantly decreased over time—interestingly, to a similar level to what previously was observed for nonspecific VEGF binding to nGB-NH_2_ and nBG-SCR.

To further assess the specificity of VEGF binding to nBG-PR1P, we used a competitive binding experiment where VEGF binding was studied in the presence of BSA at a 1:1 ratio, where the overall protein content in the immersion solution remained the same (2 μg mL^–1^). Under such competitive binding conditions, a high binding efficiency toward VEGF of about 82.9% was found. Again, no time-dependent decrease in VEGF immobilization from the complex during subsequent agitation and washing over 7 days was observed (Figure 4c). VEGF and albumin adsorption to control materials and their release was comparable to exposure to single-protein solutions (Figure 4d). Vice versa, nonspecific binding and release of albumin was not altered by the presence of VEGF. Taken together, nBG-PR1P binds VEGF specifically, and binding is not altered in the presence of a second protein. Moreover, the formed PR1P/VEGF complex is stable for up to 7 days, i.e. specific binding via PR1P allows long-term immobilization of VEGF on our nBG carriers. These findings demonstrate for the first time the immobilization of VEGF on nBG particles via a binding peptide.

### Biological Activity of Immobilized VEGF

**Figure 5:**
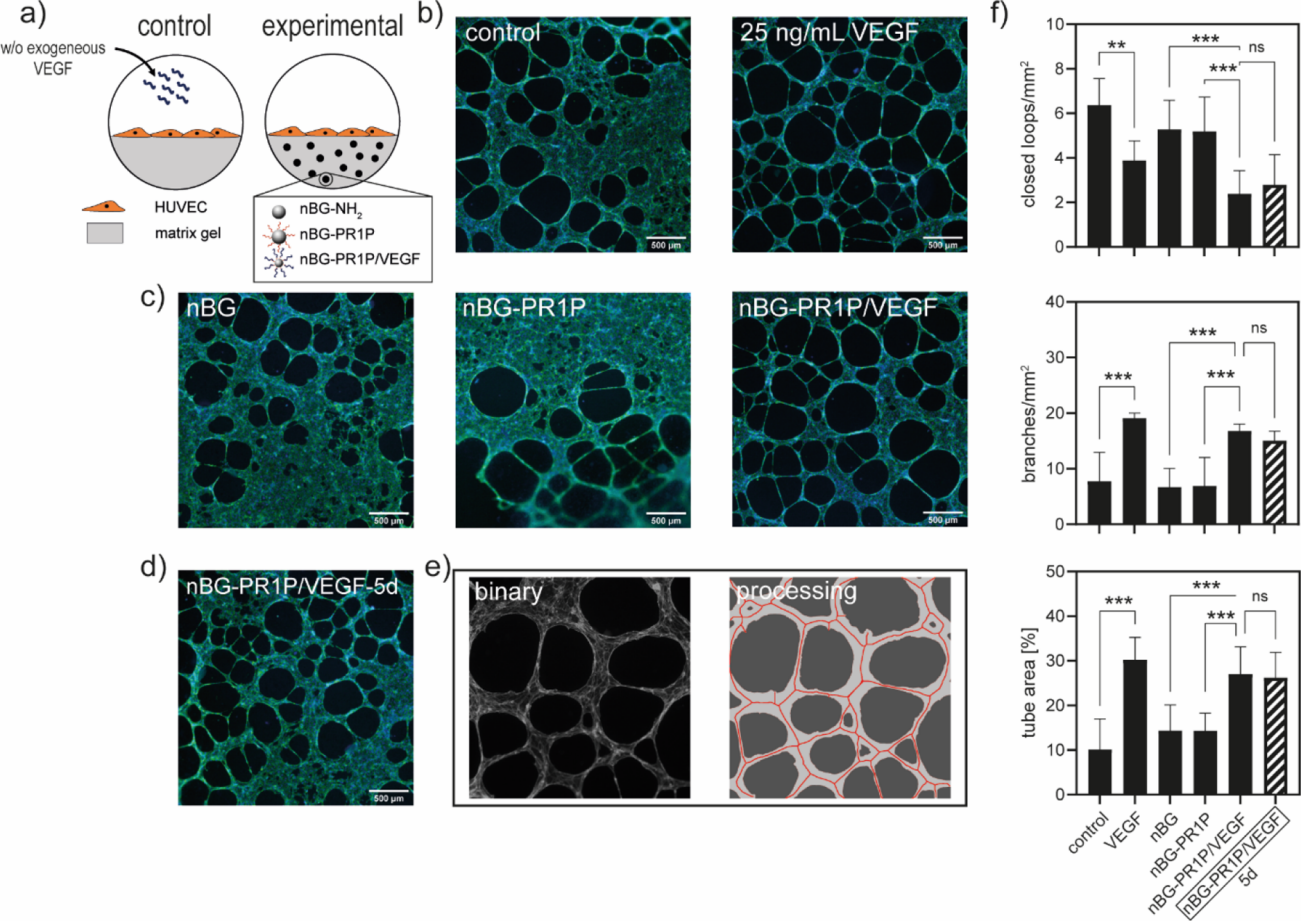
(a) Schematic showing the in vitro test setup. (b) Stimulation of in vitro tubule formation by exogenous VEGF (0 vs 25 ng mL^–1^) in HUVEC cultures on cell basement membrane matrix gel. (c) Tubule formation in response to VEGF-free material controls (nBG-NH_2_ and nBG-PR1P) and experimental nBG-PR1P/VEGF prepared directly prior to the in vitro experiment and embedded in the matrix gel. (d) Effect of nBG-PR1P/VEGF aged for 5 days prior to the start of the experiment on tubule formation. Fluorescent labeling shows F-actin (green) and nuclei (blue). Image processing (e) and quantitative analysis (f) of the tubular network in terms of closed loops, tube width, number of vascular branches, and total tube area (*P < 0.033, **P < 0.002, ***P < 0.001).

It is key for an efficient VEGF carrier system to ensure its biological activity at the site of injury. Here, the pro-angiogenic capacity of immobilized VEGF was tested in an endothelial cell tubule formation assay^62^ that has been shown to be highly predictive for the pro-angiogenic activity of various substances in vivo*.*^63^ Using a commercial basement membrane matrix gel (Geltrex, Gibco) with reduced content of pro-angiogenic GFs (in particular VEGF) as substrate to mimic the in vivo extracellular environment of vasculature-forming endothelial cells, this model allows studying of early angiogenic events.^63^ By integrating our material into the gel and providing no exogenous VEGF to the experimental groups, the cellular response to nBG-PR1P/VEGF and, therefore, the biological activity of immobilized VEGF was studied (Figure 5a). Figure 5b depicts the tubule formation of HUVEC cell control groups cultured on the gel matrix for 18 h. The addition of 25 ng mL^–1^ exogenous VEGF resulted in the formation of larger, more pronounced tubules, indicating a more mature prevascular network.^63^ Tubule formation in experimental groups with nBG-NH_2_ and nBG-PR1P contained in the matrix gel (Figure 5c) resembled the pattern seen in the VEGF-free control group. This is in line with findings from Adini et al.,^42^ which showed that the PR1P peptide alone (without VEGF) does not have an effect on angiogenesis. In contrast, a structure comparable to the control group supplemented with 25 ng mL^–1^ exogenous VEGF can be seen on the gel matrix containing nBG-PR1P/VEGF. In VEGF-treated groups (either on pure gels supplemented with exogenous VEGF or on gels containing nBG-PR1P/VEGF), loops were larger and areas where a more dense cell coverage could be observed were less pronounced. Several studies have described comparable effects of soluble as well as immobilized VEGF on HUVECs cultured on basement membrane matrices,^32,40,63^ allowing us to attribute the more pronounced network formation to the biological activity of immobilized VEGF.

In addition, it is important to note that the total amount of immobilized VEGF in the nBG-PR1P/VEGF group was about 4.3 ng per well (0.5 mg of nBG-PR1P holding 8.6 ng mg^–1^) and, therefore, about 2.5 times lower than the concentration of exogenously added VEGF. The fact that a comparable biological effect was observed nonetheless suggests that PR1P complexation enhanced the effect of VEGF, which is in accordance with earlier studies; although not fully explained to date, a potentiating^42^ and preserving^44^ effect of PR1P on VEGF has been described*.*

In solution, VEGF undergoes degradation with a half-life of around 100 min, which is further reduced under in vitro conditions.^52,58,64^ Although the time used to immobilize VEGF in this study was reduced to 3 h and was therefore considerably shorter than in many other studies that demonstrated effectiveness of released VEGF,^29,32,59^ it was 1.8 times the reported half-life of VEGF. Evidence exists that matrix immobilization can increase the half-life of VEGF.^58^ Furthermore, a stabilizing effect of PR1P has been proposed.^42−44^ Based on these findings, we hypothesized that by employing nBG-PR1P as a carrier for VEGF, its biological activity can be preserved over a longer period of time. We therefore studied the pro-angiogenic effect of nBG-PR1P/VEGF synthesized 5 days prior to the start of the cell culture experiment and stored at 37 °C in PBS under constant agitation. Interestingly, no qualitative difference in the tubule networks formed on matrices containing freshly prepared and aged nBG-PR1P/VEGF was found (Figure 4d). Quantitative analysis of the network parameter did not show a significant reduction in tubule formation in cultures treated with aged nBG-PR1P/VEGF (Figure 4f). This demonstrates that the biological activity of VEGF is not only preserved during immobilization on nBG-PR1P but is maintained over at least 5 days when immobilized on the nBG-PR1P carrier under degrading conditions. While the preservation of biological activity of VEGF immobilized via encapsulation in polymeric microspheres in frozen state has been demonstrated over several months,^24^ few studies on the long-term stability of VEGF immobilized on carriers under degrading conditions (aqueous medium, 37 °C) have been reported. Using another VEGF isoform (VEGF_121_ compared to VEGF_165_ used in this study) covalently coupled to a fibrin gel via an α_2_-PL_1–8_ peptide coupled to the N terminus of VEGF, Largo et al.^65^ have demonstrated retention of 80% biological effectiveness on HUVEC proliferation over 12 days in vitro but did not assess in vitro pro-angiogenic potency. In a second study, the same group demonstrated a pro-angiogenic effect of VEGF_164_ immobilized in fibrin gels using the same mechanism extracted from an in vivo model after 2 weeks.^66^ Therefore, to the best of our knowledge, this is the first study to demonstrate pro-angiogenic activity of VEGF noncovalently immobilized on a biomaterial carrier over at least 5 days.

## Conclusions

Insufficient neovascularization remains a limiting factor in the clinical translation of regenerative strategies. This is addressed by therapeutic angiogenesis (TA), which aims to steer neovascularization to allow faster defect regeneration and longer implant lifetime. A major strategy of TA is the targeted administration of pro-angiogenic growth factors, among which VEGF is the most prominent. However, the lack of control over dosage and spatial distribution limits their clinical use.

Here, we developed a VEGF immobilization system based on bioactive glass nanoparticles (nBGs) decorated with a peptide PR1P, which binds the pro-angiogenic growth factor VEGF_165_ with high specificity. In contrast to previously described immobilization strategies based on physical entrapment, nonspecific electrostatic interactions, or covalent coupling, our original approach utilizes specific peptide–protein interactions and therefore ensures the immobilization of VEGF in its native, biologically active state. nBGs have previously been demonstrated to stimulate bone tissue formation and degrade in a controlled manner and have been successfully integrated into biopolymer and hydrogel composites to increase their osteoregenerative potential. In this sense, the system described herein is a promising strategy in orthopedic applications that require both bone regeneration and neovascularization.

To allow VEGF binding, nBGs were surface-grafted with high control over functional groups and PR1P coupling. On PR1P-functionalized nBG particles, up to 8.5 ng mg^–1^ VEGF could be immobilized with high efficiency (>80%), specificity in the presence of a second protein, and stability with no significant release of VEGF over 7 days. High biological effectiveness of immobilized VEGF was found, which stimulated early angiogenic events in endothelial cell cultures at much lower doses than exogenously added VEGF. The biological activity of immobilized VEGF was further shown to be preserved for at least 5 days under in vitro conditions and, thus, over a multiple of the reported half-life of VEGF under aqueous conditions.

We therefore propose nBGs decorated with PR1P that bind VEGF a as novel growth factor immobilization system for local administration in large bone defect regeneration, preferably embedded in (bio)polymer composites. To the best of our knowledge, this is the first time that a growth factor-binding peptide was covalently coupled to the surface of a BG surface and used to immobilize the factor on the particle.

This approach combines a central strategy of therapeutic angiogenesis, i.e., the local administration of pro-angiogenic factors, with a well-established synthetic bone graft material (bioactive glass), prevents VEGF degradation upon immobilization, and preserves the bioactivity of the VEGF. The system therefore holds great potential to overcome the problem of insufficient neovascularization and, therefore, impaired regeneration in large bone defects.

Beyond their application in hard tissue regeneration, nBGs bear great potential in other fields of regenerative medicine, such as wound healing or nanomedicine as reviewed by Zheng and Boccaccini.^67^ As the peptide coupling reaction to amine functionalities established on the nBG is not specific to the PR1P peptide employed in this study, it appears feasible to transfer our approach to other functional or GF-targeting peptides and thereby address other regenerative processes. A growing number of functional or GF-binding peptides derived from active domains of soluble or extracellular or matrix proteins^68−70^ has been identified over the past years, allowing the design of new therapeutic approaches based on nanosized biomaterial carriers.

## Materials and Methods

### Reagents

Methanol, tetraethyl orthosilicate (TEOS, ≥99.0%), triethyl phosphate (TEP, ≥99.8%), calcium nitrate tetrahydrate (CNT, ≥98%), 3-aminopropyl triethoxysilane (APTES), albumin–fluorescein isothiocyanate conjugate (FITC albumin), *N*-(3-dimethylaminopropyl)-*N*′-ethylcarbodiimide hydrochloride (EDC), *N*-hydroxysulfosuccinimide sodium salt (sulfo-NHS), albumin–fluorescein isothiocyanate conjugate (albumin^FITC^), and 37% paraformaldehyde (PFA) were all obtained from Sigma Aldrich. Aqueous ammonia (25%) was purchased form Carl Roth. Absolute ethanol (EtOH) and phosphate-buffered saline (tablets, PBS) were purchased from VWR. NHS–Fluorescein (5/6-carboxyfluorescein succinimidyl ester) was purchased from Thermo Fisher. Human vascular endothelial growth factor (VEGF_165_) was purchased from Miltenyi.

### Binding Peptide

The PR1P peptide as well as a scrambled (SCR) form were designed based on the work of Adini et al.^42^ and modified with a 8-mer polyglycine sequence at the N terminus acting as spacer between the functional domain of the peptide and the nBG surface (Table 1). The peptides and a C-terminally FITC-tagged variant (PR1P^FITC^) were commercially synthesized by GenScript (Piscataway, USA; purity ≥ 90%).

### Material Synthesis

#### Synthesis of Nanosized Bioactive Glass (nBG) Particles

nBG particles were synthesized using a base-catalyzed sol–gel process. All synthesis steps were performed at room temperature (RT) and under vigorous stirring. In a typical batch, 2.77 g of TEOS and 0.25 mL of TEP were dissolved in 2.8 mL of methanol. After 30 min, a mixture of 12.6 mL of ddH_2_O, 29 mL of ethanol, and 2.56 mL of 25% ammonia was added. After another 60 min, 1.57 g of CNT was added. After 12 h, nBG particles were collected by centrifugation at 1500*g* for 5 min and washed in methanol and ethanol. The collected particles were vacuum-dried, calcinated at 600 °C for 3 h with a heating rate of 2 K min^–1^ (Nabertherm), and resuspended in ethanol at a concentration of 10 mg mL^–1^ for subsequent processing.

#### Amine Modification

Aliquots of nBG were dispersed in ethanol at 2 μg mL^–1^ in 100% ethanol and heated for 30 min to 80 °C under reflux while flushing with nitrogen. Then, appropriate amounts of APTES were added to achieve an APTES/nBG ratio of 4.2 or 42 μmol mg^–1^, respectively. After reaction for 3 h, the reaction was cooled to RT, and nBG-NH_2_ particles were collected by centrifugation at 1500*g* for 5 min, washed twice with ethanol, and resuspended at 10 mg mL^–1^ in ethanol.

#### Peptide Modification

Peptide aliquots (RR1P, SCR; 100 μg mL^–1^) were activated in phosphate-buffered saline (PBS, pH = 7.0) by incubation with EDC (0.6 mmol mL^–1^) and sulfo-NHS (0.6 mmol mL^–1^) for 30 min under continuous shaking at RT. Coupling to nBG-NH_2_ was performed at a nBG concentration of 20 mg mL^–1^ in PBS (pH = 7.0) by adding appropriate amounts of activated peptide and incubating for 24 h under continuous shaking at RT. As-synthesized nBG served as a control to assess nonspecific binding of the peptide to nBG. Peptide/nBG ratios of 0.2, 1.0, 5.0, 25.0, and 50.0 μg mg^–1^ were used. Peptide-functionalized particles were collected by gentle centrifugation (800*g*, 10 min), and unbound peptide was removed by double-washing in PBS. Functionalized particles were suspended at 10 mg mL^–1^ in PBS (pH = 7.4) and stored at 4 °C for a maximum of 7 days.

### Material Characterization

#### Nanoparticle Characterization

X-ray diffractograms (XRD) of as-synthesized nBG were recorded using a Bruker D2 Phaser diffractometer using Cu K_α_ radiation (λ = 1.5406 Å) in the range of 6 ≤ 2Θ ≤ 60° in increments of 0.02° and an integration time of 0.75 s. Functional groups were assessed using attenuated total reflection Fourier transform-infrared spectroscopy (ATR-FTIR, Nicolet iS50) running 32 scans between 400 and 4000 cm^–1^ with a resolution of 0.5 cm^–1^. Spectra were evaluated using Spectragryph (F. Menges, Version 12, 2018, http://effemm2.de/spectragryph/). nBG morphology and size were assessed using transmission electron microscopy (TEM, FEI Tecnai G2 Spirit BioTWIN iCorr (G0.201)), and particle size was determined using ImageJ (ImageJ 1.52n). Surface charges of as-synthesized and modified particles suspended in PBS (0.5 mg mL^–1^) were further assessed using zeta potential analysis (Malvern Zetasizer Nano, Panalytical, UK).

#### Amine Modification

nBG-NH_2_ was dispersed in absolute ethanol at 5 mg mL^–1^ and reacted with 63 μg mL^–1^ NHS-FITC during overnight stirring.^57^ Labeled nBG-NH_2_ was collected by centrifugation (1500*g*, 5 min) and washed three times with absolute ethanol to remove unbound dye. Samples were redispersed in absolute ethanol at 5 mg mL^–1^ and fluorescence (λ_ex_ = 488–14 nm, λ_em_ = 535–30 nm) of 100 μL aliquots (*n* = 4) was quantified using a ClARIOstar spectrophotometer (BMG LABTECH, Germany). Unstained nBG-NH_2_ and unmodified nBG that underwent the same protocol served as sample blank and negative control, respectively.

#### Peptide Functionalization

The peptide functionalization of nBG-NH_2_ particles was quantitatively assessed by fluorescence intensity measurement. Particles were functionalized with FITC-labeled peptide (PR1P^FITC^) as described above, washed five times with PBS (pH = 7.4), collected by gentle centrifugation (800*g*, 10 min), and resuspended at 20 mg mL^–1^ in PBS. Fluorescence was measured (λ_ex_ = 483–14 nm, λ_em_ = 530–30 nm) and evaluated using a standard curve prepared from PR1P^FITC^ (Figure S2a).

#### VEGF Loading and Competitive Binding

VEGF was loaded onto nBG particles by immersion of 20 mg mL^–1^ nBG-PR1P in PBS (pH 7.4) containing either 0.02 or 0.2 μg mL^–1^ VEGF for 3 h under continuous stirring at RT. Subsequently, loaded particles were collected by centrifugation (1000*g*, 5 min) and resuspended in PBS at 10 mg mL^–1^. To quantitatively assess GF loading, VEGF was labeled with Alexa Fluor 647 using the Lightning-Link Alexa Fluor 647 Conjugation Kit (Abcam) according to the manufacturer’s instructions (VEGF^647^). Fluorescence intensity (λ_ex_ = 625–30 nm, λ_em_ = 680–30 nm) was measured in suspension and evaluated against a standard curve prepared from VEGF^647^ to quantitatively assess bound VEGF (Figure S2b). Long-term stability of the VEGF loading was tested by immersing loaded particles for 1, 3, and 7 days in PBS (pH 7.4) under continuous shaking, followed by an additional washing step in PBS and fluorescence measurement at each time point.

Nonspecific and competitive binding were tested by immersion of nBG-PR1P in 0.1 μg mL^–1^ albumin^FITC^ in PBS as well as PBS containing both 0.1 μg mL^–1^ albumin^FITC^ and 0.1 μg mL^–1^ VEGF^647^, respectively, for 3 h. Binding of albumin^FITC^ was quantified by fluorescence intensity (λ_ex_ = 483–14 nm, λ_em_ = 530–30 nm) and evaluated using a calibration line prepared from pure albumin^FITC^ following the washing and immersion regimen described above.

#### In Vitro Tube Formation Assay

Human umbilical vein endothelial cells (HUVEC) pooled from different donors (Lonza) and cultured without VEGF supplementation were cultured in endothelial growth medium (EGM, Lonza) at 37 °C and 5% CO_2_. Cells of the fourth passage were used for in vitro experiments.

An in vitro tube formation assay was performed in 48-well tissue culture plates (NUNC) coated with 50 uL per well Geltrex (Thermo Fisher Scientific) according to the manufacturer’s instructions. To assess the effect of nBG particles on tubule formation as well as the biological activity of VEGF loaded onto nBG-PR1P as described above, (modified) particles were suspended into Geltrex prior to coating at a concentration of 0.5 mg per well. Loading of VEGF onto nBG-PR1P was performed over 3 h as described above directly before the start of the in vitro experiment. To test the long-term biological activity of immobilized VEGF, nBG-PR1P/VEGF that was stored in PBS at RT under constant agitation for 5 days prior to the start of the experiment were included. Non-VEGF-loaded nBG-PR1P as well as nonfunctionalized nBG-NH_2_ served as material controls. HUVECs cultured in the absence of materials and without 25 ng mL^–1^ VEGF served as material-free controls.

HUVECs were trypsinized at approximately 80% confluency and seeded at a density of 4 × 10^4^ cells cm^–2^ in EGM (*n* = 3; 500 μL per well). After 18 h incubation at 37 °C and 5% CO_2_, cells were washed twice with PBS, fixed with 300 μL of 4% paraformaldehyde (PFA) per well for 30 min, and stored in 0.4% PFA at 4 °C for subsequent analysis.

#### Fluorescent Staining and Microscopy

Fixed cells were permeabilized with 0.1% Triton X-100 in PBS (200 uL per well) for 10 min and washed twice with PBS. Then, F-actin staining was performed by incubation with 1:200 PBS-diluted Alexa Fluor 568 phalloidin (Thermo Fisher Scientific; 50 μL per well) for 20 min in the dark followed by two washing steps in PBS. Cell nuclei were stained by immersion in DAPI (Thermo Fisher Scientific; 50 μL per well) at a concentration of 0.5 μg mL^–1^ in PBS for 5 min followed by a last washing step. Imaging was performed using an inverted Nikon TiS/L100 microscope equipped with a Nikon DS-Ri2 camera (operated in monochromatic mode) and a Lumencor Sola SE II for fluorescence. Images were processed and analyzed using Fiji (ImageJ 1.53c), and quantitative analysis of in vitro tubule formation was performed using the AutoTube plugin^71^ for MATLAB (R2018a).

#### Statistical Analysis

Unless stated differently, all experiments were performed using triplicates (*n* = 3) and data is presented as mean ± standard deviation. Statistical evaluation was performed using GraphPad Prism 9.1 using two-way analysis of variance (ANOVA) with post hoc testing (Bonferroni method), and mean differences were considered statistically significant for *P* < 0.033.

## Supporting Information Available

Characterization of nBG and nBG-NH_2_ (XRD and FTIR); calibration data for PR1P and VEGF quantification (PDF)
