# Supplementary material for: Peptide-Modified Nano-Bioactive Glass for Targeted Immobilization of Native VEGF
Source: ACS Appl Mater Interfaces. 2022 Jan 18;14(4):4959–68. doi: 10.1021/acsami.1c21378 (PMC8815037; doi:10.1021/acsami.1c21378)
Supplement: Supplementary file 1 — am1c21378_si_001.pdf [file am1c21378_si_001.pdf]

Supporting information

Peptide-modified nano-bioactive glass for targeted immobilization of native VEGF

Matthias Schumacher[1*], Pamela Habibović[1], Sabine van Rijt[1]

[1]Department of Instructive Biomaterials Engineering, MERLN Institute for Technology-Inspired Regenerative Medicine, Maastricht University, 6229 ER Maastricht, The Netherlands

*Email: m.schumacher@maastrichtuniversity.nl

As-synthesized nBG as well as amine-functionalized nBG (nBG-NH_2) were shown to be fully amorphous by XRD (Fig. S1 a). FTIR analysis showed characteristic absorption bands around 1040 cm^{-1} and 800 cm^{-1} that are characteristic for SiO_2-based glasses and at 568 and 606 cm^{-1} associated with PO_4^{3-} and H_3PO_4 groups, respectively.[54]

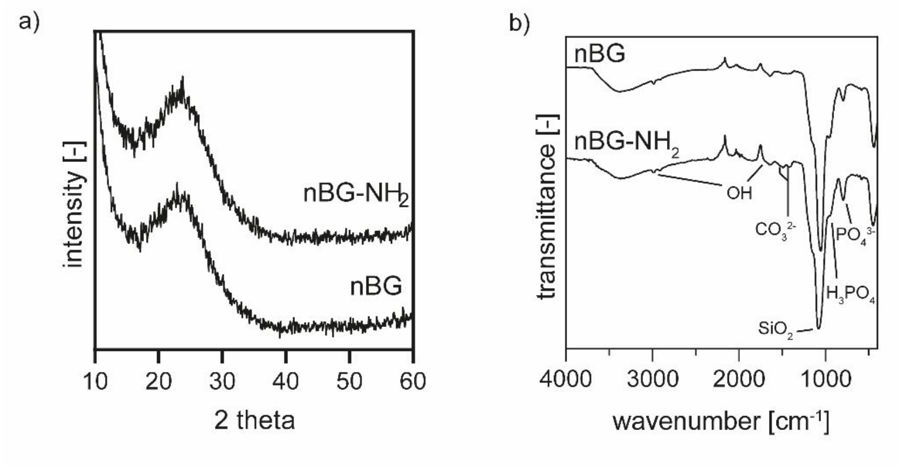

Figure S1: XRD diffractograms (a) and FTIR-spectra (b) of nBG and nBG-NH_2.

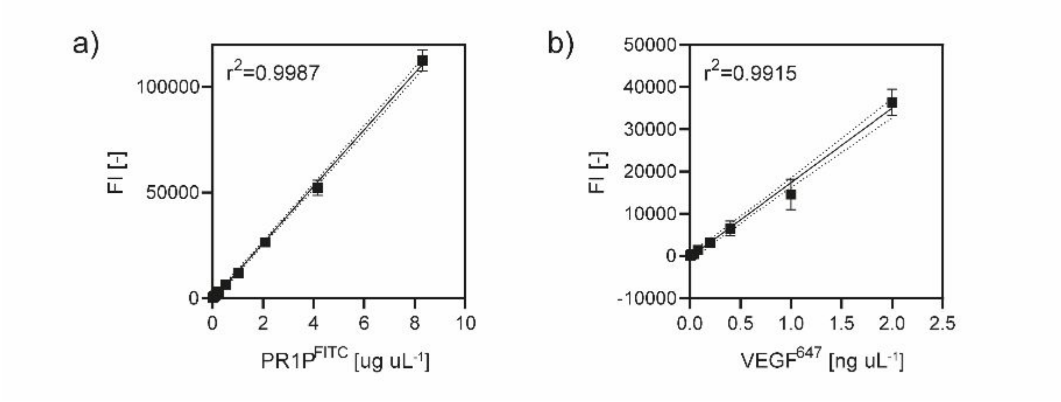

Figure S2: Calibration used for quantitative evaluation of PR1P binding to nBG (a) as well as VEGF immobilization on nBG-PR1P (b) recorded using fluorescent-tagged peptide and GF.
